# Associated-Onset Symptoms and Post-COVID-19 Symptoms in Hospitalized COVID-19 Survivors Infected with Wuhan, Alpha or Delta SARS-CoV-2 Variant

**DOI:** 10.3390/pathogens11070725

**Published:** 2022-06-25

**Authors:** César Fernández-de-las-Peñas, Ignacio Cancela-Cilleruelo, Jorge Rodríguez-Jiménez, Victor Gómez-Mayordomo, Oscar J. Pellicer-Valero, José D. Martín-Guerrero, Valentín Hernández-Barrera, Lars Arendt-Nielsen, Juan Torres-Macho

**Affiliations:** 1 Department of Physical Therapy, Occupational Therapy, Physical Medicine and Rehabilitation, Universidad Rey Juan Carlos (URJC), 28922 Madrid, Spain; ignacio.cancela@urjc.es (I.C.-C.); jorge.rodriguez@urjc.es (J.R.-J.); 2 CNAP, Center for Sensory-Motor Interaction (SMI), Department of Health Science and Technology, Faculty of Medicine, Aalborg University, 9220 Aalborg, Denmark; lan@hst.aau.dk; 3 Department of Neurology, Hospital Clínico San Carlos, 28040 Madrid, Spain; vicmayordomo@gmail.com; 4 Intelligent Data Analysis Laboratory, Department of Electronic Engineering, ETSE (Engineering School), Universitat de València (UV), 46010 Valencia, Spain; oscar.pellicer@uv.es (O.J.P.-V.); jose.d.martin@uv.es (J.D.M.-G.); 5 Department of Public Health, Universidad Rey Juan Carlos (URJC), 28922 Madrid, Spain; valentin.hernandez@urjc.es; 6 Department of Medical Gastroenterology, Aalborg University Hospital, 9100 Aalborg, Denmark; 7 Department of Internal Medicine, Hospital Universitario Infanta Leonor-Virgen de la Torre, 28031 Madrid, Spain; juan.torresm@salud.madrid.org; 8 Department of Medicine, School of Medicine, Universidad Complutense de Madrid, 28040 Madrid, Spain

**Keywords:** COVID-19, post-COVID-19, variant, delta, alpha, Wuhan, risk factors

## Abstract

This study compared associated-symptoms at the acute phase of infection and post-COVID-19 symptoms between individuals hospitalized with the Wuhan, Alpha or Delta SARS-CoV-2 variant. Non-vaccinated individuals hospitalized because of SARS-CoV-2 infection in one hospital during three different waves of the pandemic (Wuhan, Alpha or Delta) were scheduled for a telephone interview. The presence of post-COVID-19 symptoms was systematically assessed. Hospitalization and clinical data were collected from medical records. A total of 201 patients infected with the Wuhan variant, 211 with the Alpha variant and 202 with Delta variant were assessed six months after hospitalization. Patients infected with the Wuhan variant had a greater number of symptoms at hospital admission (higher prevalence of fever, dyspnea or gastrointestinal problems) than those infected with Alpha or Delta variant (*p* < 0.01). A greater proportion of patients infected with the Delta variant reported headache, anosmia or ageusia as onset symptoms (*p* < 0.01). The mean number of post-COVID-19 symptoms was higher (*p* < 0.001) in individuals infected with the Wuhan variant (mean: 2.7 ± 1.3) than in those infected with the Alpha (mean: 1.8 ± 1.1) or Delta (mean: 2.1 ± 1.5) variant. Post-COVID-19 dyspnea was more prevalent (*p* < 0.001) in people infected with the Wuhan variant, whereas hair loss was higher in those infected with the Delta variant (*p* = 0.002). No differences in post-COVID-19 fatigue by SARS-CoV-2 variant were found (*p* = 0.594). Differences in COVID-19 associated onset symptoms and post-COVID-19 dyspnea were observed depending on the SARS-CoV-2 variant. The presence of fatigue was a common post-COVID-19 symptom to all SARS-CoV-2 variants.

## 1. Introduction

The severe acute respiratory syndrome coronavirus 2 (SARS-CoV-2), the agent causing the coronavirus disease, 2019 (COVID-19), has change the world in the last two years. The quick spreading of SARS-CoV-2 virus across the world has been favored by the appearance of several variants such as Alpha, Beta, Gamma, Delta, Epsilon, Zeta, Eta, Theta, Lota, Kappa, Lambda and, more recently, Omicron [1]. Among all these variants, Alpha (B.1.1.7), Beta (B.1.351), Gamma (P.1), Delta (B.1.617.2) and Omicron (B.1.1.529/BA.1) have been considered the variants of concern (VOCs), in addition to the Wuhan variants [2]. For instance, Alpha variant had more viral load than the Wuhan variants, Delta exhibits more viral load than Alpha variant [3], whereas Omicron variant revealed the highest level of transmissibility [4]. Nevertheless, just higher viral load is not the only factor explaining the increased infectivity of these variants of concern [5]. In addition to a higher transmissibility, escape from vaccines and the possibility of reinfections have been different topics of concern and extensive research of SARS-CoV-2 variants.

Monitoring the epidemiology and clinical manifestations of SARS-CoV-2 variants are highly relevant for better identification, management and control. Common symptoms reported in people with COVID-19 at the acute phase are fever, dyspnea and cough, while minor symptoms include dysgeusia, anosmia, gastrointestinal symptoms, headache and skin lesions [6,7,8]. However, there is preliminary evidence supporting that clinical manifestations and severity of COVID-19 are different according to SARS-CoV-2 variant [9,10]. Although some studies have investigated differences in onset-associated symptoms between some variants of concern, most of them have focused on the Omicron variant. Further studies systematically comparing these data on Alpha and Delta variants are needed.

Another important clinical aspect is the development of symptoms after the acute phase of the infection, called long COVID-19 [11] or post-COVID-19 [12]. Evidence supports that 60% of COVID-19 survivors will experience post-COVID-19 symptoms at least during the first year after the infection [13,14]. Fatigue, dyspnea, musculoskeletal pain and headache are the most prevalent post-COVID-19 symptoms, but also others such as ageusia, anosmia or skin rashes are present [13,14]. In fact, the presence of post-COVID-19 symptoms is associated with worse health-related quality of life [15]. Most studies investigating the presence of post-COVID-19 symptoms had included COVID-19 survivors infected with the Wuhan variant. Due to the fact that millions of people will experience long COVID-19 in the future [16], understanding the association of long COVID-19 with the different SARS-CoV-2 variants is clearly needed. Preliminary data suggest that SARS-CoV-2 variants may induce different long COVID-19 phenotypes [17]; however, no published study to date has systematically investigated the differences in post-COVID-19 symptoms depending on the SARS-CoV-2 variant. The aims of this study were: (1) to compare clinical features and associated symptoms at the acute phase of the infection; and (2) to compare the development of post-COVID-19 symptoms between subjects hospitalized with the Wuhan, Alpha or Delta SARS-CoV-2 variant during different waves of the COVID-19 pandemic.

## 2. Methods

### 2.1. Participants

The current study included individuals who were hospitalized due to acute SARS-CoV-2 infection (ICD-10 code) from one urban hospital in Madrid, Spain. The diagnosis of SARS-CoV-2 infection was confirmed at hospital admission with real-time reverse transcription-polymerase chain reaction (RT-PCR) assay of nasopharyngeal/oral swab samples and the presence of both clinical and radiological findings. The RT-PCR positive nasopharyngeal/oral samples were screened to assess the SARS-CoV-2 viral lineage to determine the infected variant. For that purpose, sanger sequencing of the receptor binding domain (RBD) was used to determine the SARS-CoV-2 variant type in all patients hospitalized during the third (Alpha) and fifth (Delta) waves.

Patients included from the first wave (March–April 2020) presented the Wuhan variant since no other variant of concern was present at that time. For the third (February–March 2021) and fifth (July–August 2021) waves, only those patients presenting with the Alpha (B.1.1.7) or Delta (B.1.617.2) variant in the sequencing were included. Due to the appearance of vaccines in January 2021, we included individuals who had not received any dose of vaccination before infection and hospitalization. Additionally, patients reporting re-infections were also excluded. All hospitalized COVID-19 survivors discharged during each wave of the pandemic (approximately 450 on each wave) were included in an anonymous database and a random selection of 250 patients from each wave were selected using online randomization software. The study was approved by the Local Ethic Committee of the Hospital (URJC0907202015920, HUIL/092-20). All participants provided informed consent before collecting any data.

### 2.2. Procedure

Demographic (age, gender, height, weight), clinical (COVID-19 associated onset symptoms at hospital admission, pre-existing medical comorbidities) and hospitalization (intensive care unit (ICU) admission, days at hospital) data were collected from hospital medical records.

Participants who agreed to participate were scheduled for a telephone interview by trained researchers. Participants were asked to report the presence/absence of symptoms appearing after hospitalization and whether the symptoms persisted at the time of the study. A predefined list of post-COVID-19 symptoms including dyspnea, fatigue, anosmia, ageusia, hair loss, chest pain, palpitations, diarrhea, skin rashes, brain fog, visual problems (e.g., worsening of vision, blurred vision), cough and loss of concentration was systematically assessed. Further, patients were free to report any symptom that they suffered from and considered relevant.

### 2.3. Statistical Analysis

Data are presented as mean (standard deviation, SD) or number of cases (percentage) as appropriate. We compared the differences in COVID-19 associated-onset symptoms at hospitalization and the presence of post-COVID-19 symptoms by SARS-CoV-2 variant with Chi-squared or one-way-ANOVA tests as needed. The level of significance was set at 0.05, with *p*-values from all tests being corrected by means of the Holm-Bonferroni correction. Data were collected with STATA 16.1 and processed using Python’s library pandas 0.25.3; Scipy 1.5.2 was employed for conducting the statistical tests and statsmodels 0.11.0 for performing *p*-value correction.

## 3. Results

From 250 patients randomly selected to participate during each wave, 201 (mean age: 60.5, SD: 10.5 years, 54.2% women) individuals infected with the Wuhan variant, 211 (mean age: 70.0, SD: 15.5 years, 51.1% women) infected with the Alpha variant and 202 (mean age: 56.5, SD: 21.0 years, 54.4% women) with the Delta variant fulfilled all criteria and agreed to participate. All participants were assessed at a similar follow-up period after hospital discharge: Wuhan (mean: 6.5, SD: 1.0 months), Alpha (mean: 6.0, SD: 1.2 months) or Delta (mean: 6.3, SD: 1.0 months) variant.

**Figure 1:**
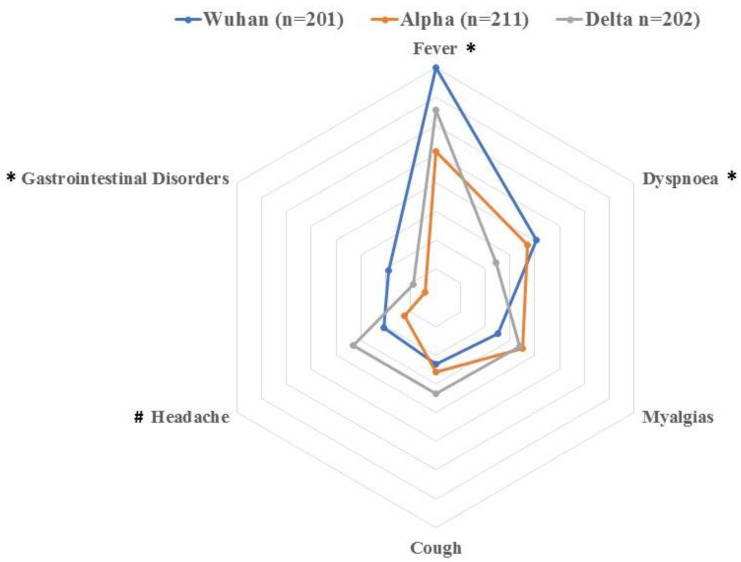
Distribution of the most prevalent COVID-19 associated-onset symptoms (fever, dyspnea, myalgia, cough, headache and gastrointestinal problems) in individuals infected with the Wuhan, Alpha or Delta variant. Each surrounding hexagon represents 10% of percentage of prevalence. * Significant differences between the Wuhan variant versus Alpha and Delta variants (*p* < 0.01). # Significant differences between the Delta variant versus Wuhan and Alpha variants (*p* < 0.001).

The features of the study population by SARS-CoV-2 variant are summarized in Table 1. Overall, patients infected with the Alpha variant were older and showed a longer stay of hospitalization (both, *p* < 0.001) than those subjects infected with Wuhan or Delta variant. The most common symptoms associated with SARS-CoV-2 infection at hospital admission were fever (assessed on the arm pit with a standard thermometer and defined when <37.5 °C), dyspnea, myalgia and cough. Patients infected with the Wuhan variant showed a greater number of onset symptoms at hospitalization, with a greater prevalence of subjects experiencing fever, dyspnea or gastrointestinal problems (*p* < 0.01) than those infected with Alpha or Delta variant (Table 1). Further, a greater proportion of patients infected with the Delta variant exhibited headache, anosmia and ageusia as onset symptoms at hospital admission (all, *p* < 0.01, Figure 1).

**Figure 2:**
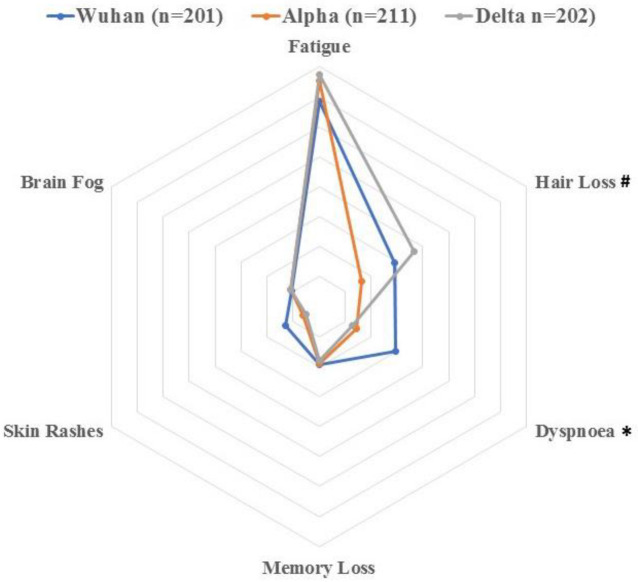
Distribution of the most prevalent post-COVID-19 symptoms (fatigue, dyspnoea, hair loss, memory loss, skin rashes and brain fog) in individuals infected with the Wuhan, Alpha or Delta variant. Each surrounding hexagon represents 10% of percentage of prevalence. * Significant differences between the Wuhan variant versus Alpha and Delta variants (*p* < 0.01). # Significant differences between the Delta variant versus Wuhan and Alpha variants (*p* < 0.001).

The most prevalent post-COVID-19 symptoms at 6 months after hospital discharge were fatigue, dyspnea and hair loss. The mean number of post-COVID-19 symptoms was higher in subjects infected with the Wuhan variant as compared with people infected with Alpha or Delta variant (*p* < 0.001, Table 2). The presence of post-COVID-19 dyspnea was significantly higher (*p* < 0.001) in individuals infected with the Wuhan variant (Figure 2), whereas the presence of hair loss was higher in those infected with the Delta variant (*p* = 0.002). No significant differences in the presence of post-COVID-19 fatigue depending on the SARS-CoV-2 variant were identified (*p* = 0.594, Table 2).

## 4. Discussion

To the best of the author’s knowledge, this is the first published study to date systematically investigating the differences in COVID-19 associated-onset symptoms and post-COVID-19 symptoms in previously hospitalized COVID-19 survivors infected by different SARS-CoV-2 variants of concern. Some differences in COVID-19 associated onset symptoms were observed depending on the SARS-CoV-2 variant with more severe onset symptoms, (e.g., fatigue, dyspnea) in people infected with the Wuhan variant and more neurological symptoms in people infected with Delta variant. The presence of post-COVID-19 symptoms six months after hospitalization, particularly dyspnea, was also higher with the Wuhan variant. The presence of post-COVID-19 fatigue was similar with all SARS-CoV-2 variants.

We observed some differences in COVID-19 associated-onset symptoms at hospital admission between the three SARS-CoV-2 variants of concern investigated. Fever, dyspnea and gastrointestinal problems were more prevalent with the Wuhan variant and neurological symptoms (e.g., anosmia, ageusia and headache) were more prevalent with Delta variant. A previous study found that myalgia was more common in individuals infected with the Delta variant than with other variants in South Korea [10]. In our sample, the prevalence of myalgia as an onset symptom was also higher in those infected with the Delta variant, but differences did not reach statistical significance. On the contrary, Mattiuzzi et al. reported no differences in onset-associated symptoms between these variants of concern; however, these authors analyzed Web searches of the general population [18]. Few studies have systematically compared differences in onset symptoms between SARS-CoV-2 variants [9,10]. It should be noted that the presence of taste (ageusia) and smell (anosmia) disorders is a common finding in all SARS-CoV-2 variants but with a higher prevalence within the Delta variant. In fact, ageusia and anosmia have been used as a useful tool for clinical triage of COVID-19 against other respiratory infections during the pandemic [19]. These results support the assumption that COVID-19 caused by Wuhan, Alpha or Delta variant can be differentiated from flu-like symptoms caused by the influenza virus. This could be different in people infected with the Omicron variant where flu-like symptoms, e.g., sneezing or cough, are more prevalent [20]. Nevertheless, the fact that SARS-CoV-2 variants share symptoms with common flu does not mean that we should considered COVID-19 as a flu caused by the influenza virus [21].

Another finding of this study was that the prevalence of obese patients was higher in the group infected by the Delta variant when compared with Wuhan or Alpha variants. Hoang et al. also observed that patients infected during a wave where Alpha (and Beta) variants were dominant in France were more frequently obese than during the first wave associated with the Wuhan variant [9]. Although obesity is associated with a higher risk of severe COVID-19 and in-hospital mortality [22], knowledge and application of effective treatments at the acute phase of the infection during consecutive waves of the COVID-19 pandemic (Delta against Wuhan) could lead to a reduction in mortality and more survival in-hospital rates of obese patients.

Prevalent rates of most post-COVID-19 symptoms found six months after hospitalization by different SARS-CoV-2 variants agree with current meta-analyses including only individuals infected with the Wuhan variant [13,14], suggesting that post-COVID-19 symptomatology represents a common finding. A first finding of our study was that the prevalence of post-COVID-19 fatigue (70%) was similar between the three SARS-CoV-2 variants suggesting that COVID-19 is associated with long-lasting post-fatigue, sharing common symptoms with myalgic encephalomyelitis/chronic fatigue syndrome [23]. In fact, similar endothelial dysfunction has been found in patients with post-COVID-19 symptoms and those with myalgic encephalomyelitis/chronic fatigue syndrome [24]. It could be hypothesized that although some differences (e.g., higher viral load, higher transmissibility, potential reinfections) between SARS-CoV-2 variants have been identified, the pathogenic cell-to-cell mechanisms associated with the development of post-COVID-19 fatigue may be similar between all variants.

In addition, we observed that people infected with the Wuhan variant reported a greater number of post-COVID-19 symptoms, particularly dyspnea, than those infected with Alpha or Delta variant. The presence of post-COVID-19 respiratory symptoms, e.g., fatigue and dyspnoea, is associated with higher burden [25]. Accordingly, these findings agree with the assumption that coronavirus epidemics left survivors with fatigue, persistent dyspnea and burden [26]. Nevertheless, it could be expected that post-COVID-19 symptoms from individuals infected with the Wuhan variant would lead to higher burden and health costs than those infected by other variants. Obviously, we cannot exclude the hypothesis that differences found between the Wuhan and Alpha/Delta variants would be influenced by emotional and social factors surrounding the COVID-19 outbreak of each wave. For instance, several COVID-19 outbreak associated-factors, e.g., social alarm, somatization, post-traumatic stress disorder, fear or uncertainty about medical prognosis, were more pronounced during the wave associated with the Wuhan variant due to its association with a worldwide lockdown and the beginning of the pandemic than in those waves associated with Alpha or Delta variants. Nevertheless, this would be unlikely for explaining the prevalence of post-COVID-19 fatigue or dyspnea.

Although this is the first study investigating differences in COVID-19 associated onset symptoms and post-COVID-19 symptoms in three SARS-CoV-2 variants of concern, our data should be considered according to their limitations. First, our results can only be applicable to previously hospitalized COVID-19 survivors. Data according to the SARS-CoV-2 variant in non-hospitalized patients is lacking. In fact, we did not collect laboratory biomarkers, data about COVID-19 severity at hospital admission or treatments received during hospitalization, which could help to elucidate if these variables are associated with post-COVID-19 symptoms on each SARS-CoV-2 variant. There is some evidence suggesting that administration of some medications, e.g., remdesivir, at hospitalization can reduce the risk of long COVID-19 [27]. Second, current data are based on individuals who did not receive any vaccine dose and were suffering from their first infection. Preliminary evidence suggests that vaccination is able to reduce post-COVID-19 symptoms [28], but data are controversial. Similar, reinfections could induce a certain degree of immunity leading to potential decrease in post-COVID-19 symptoms. Third, we collected data through telephonic interviews, a procedure with a potential bias in population-based studies, but commonly used in post-COVID-19 research [13,14]. Fourth, the cross-sectional design of the study does not allow to determine the evolution of post-COVID-19 symptoms, making it difficult to exclusively attribute the presence of symptoms at six months after hospitalization to SARS-CoV-2. Finally, we recognize that we did not systematically assess the lineage during the first wave. Although the Wuhan variant was the dominant variant in the world until January 2021 when Alpha started to be the dominant variant, small mutations in some spike proteins led to different lineages [29], which were not investigated in the current study. Similarly, we did not collect data from the dominant variant at this moment, i.e., Omicron. Future studies including patients infected with Omicron will help to elucidate the natural evolution of COVID-19 associated-onset and post-COVID-19 symptoms.

## 5. Conclusions

The current study identified some differences in COVID-19 associated onset symptoms depending on the SARS-CoV-2 variant. People infected with the Wuhan variant showed more severe onset symptoms, (e.g., fatigue, dyspnea) whereas people infected with Delta variant exhibited more neurological symptoms (e.g., headache, anosmia or ageusia). The presence of post-COVID-19 symptoms six months after hospital discharge was higher in people infected with the Wuhan variant, particularly dyspnea. The presence of post-COVID-19 fatigue was similar with all SARS-CoV-2 variants. As a remark, evidence supports that long COVID-19 will require specific management attention independently of the SARS-CoV-2 variant.

## Figures and Tables

**Table 1:** Clinical and hospitalization data by SARS-CoV-2 variant.

	Wuhan	Alpha	Delta	*p* Value
Age (years) *	60.5 (15.5)	70.0 (15.5)	56.5 (21.0)	<0.001
**Female (%)**	109 (54.2%)	108 (51.2%)	110 (54.5%)	0.878
**Weight (kg)**	75.0 (14.0)	75.5 (16.5)	77.0 (13.5)	0.576
**Height (cm)**	168 (14)	165 (12)	166 (10)	0.254
Number of onset symptoms *	2.4 (0.8)	2.0 (1.0)	2.5 (0.8)	<0.001
**Symptoms at Admission, *n* (%)**				
Fever (>37.5 °C) *	160 (79.6%)	102 (48.3%)	131 (64.8%)	0.01
Dyspnoea *	81 (40.3%)	74 (35.1%)	49 (24.2%)	0.015
**Myalgia**	50 (24.9%)	70 (33.2%)	68 (33.7%)	0.199
**Cough**	46 (22.9%)	52 (24.6%)	67 (33.2%)	0.102
Headache *	42 (20.9%)	25 (11.85%)	66 (32.65%)	0.001
Gastrointestinal *	38 (18.9%)	8 (3.4%)	18 (8.9%)	<0.001
Anosmia *	20 (9.9%)	5 (2.4%)	29 (14.3%)	0.008
Ageusia *	13 (6.4%)	6 (2.8%)	32 (15.8%)	0.002
**Throat Pain**	10 (5.0%)	23 (10.9%)	33 (16.3%)	0.109
**Medical Co-Morbidities**				
**Hypertension**	63 (31.3%)	83 (39.3%)	72 (35.6%)	0.396
**Diabetes**	25 (12.4%)	21 (10%)	28 (13.9%)	0.501
**Cardiovascular**	32 (15.9%)	43 (20.4%)	27 (13.4%)	0.208
**Rheumatological**	3 (1.5%)	1 (0.5%)	1 (0.5%)	0.423
**Asthma**	11 (5.4%)	11 (5.2%)	19 (9.4%)	0.186
**COPD**	12 (6.0%)	14 (6.6%)	12 (5.9%)	0.949
Obesity (BMI ≥ 30) *	8 (4.0%)	19 (9.0%)	53 (26.2%)	<0.001
Cancer *	27 (13.4%)	90 (42.5%)	35 (17.3%)	<0.001
Days at hospital *^,^#	10 (13)	12 (21)	7 (8)	<0.001
**ICU admission**				
**Yes *n* (%)**	20 (9.9%)	33 (15.6%)	19 (9.4%)	0.121
**Days at ICU**	13.5 (11)	14.3 (16)	10.5 (7)	0.684

Data are expressed as mean (SD) or number (percentage) except age, which is expressed as mean (standard deviation), and days at hospital, which are expressed as median (IQR). # The comparison was conducted with the non-parametric Kruskal Wallis Test due to a non-normal distribution of the data. COPD: Chronic obstructive pulmonary disease; ICU: Intensive care unit. * Significant differences between SARS-CoV-2 variants (*p* < 0.05).

**Table 2:** Post-COVID-19 symptoms by SARS-CoV-2 variant.

	Wuhan	Alpha	Delta	*p* Value
Number of Post-COVID-19 Symptoms *	2.7 (1.3)	1.8 (1.1)	2.1 (1.5)	<0.001
**Post-COVID-19 Symptoms, *n* (%)**				
**Fatigue**	137 (68.2%)	151 (71.5%)	155 (76.35%)	0.594
Dyspnoea *	59 (29.35%)	29 (13.75%)	26 (12.8%)	<0.001
Hair loss *	58 (28.9%)	33 (15.7%)	73 (36.15%)	0.002
**Memory loss**	39 (19.4%)	38 (18.0%)	36 (17.8%)	0.921
**Skin Rashes**	26 (12.9%)	12 (5.7%)	10 (5.0%)	0.252
**Brain fog**	21 (10.4%)	22 (10.4%)	22 (10.9%)	0.989
**Attention Disorders**	14 (7.0%)	13 (6.1%)	6 (3.0%)	0.186
**Diarrhoea**	15 (7.4%)	11 (5.2%)	30 (15.0%)	0.766
**Tachycardia**	3 (1.4%)	7 (3.3%)	8 (3.95%)	0.323
**Visual Problems**	5 (2.5%)	11 (5.2%)	9 (4.5%)	0.370
**Ageusia**	10 (5.0%)	9 (4.2%)	10 (5.0%)	0.931
**Anosmia**	3 (1.5%)	12 (5.7%)	12 (6.0%)	0.654
**Cough**	3 (1.5%)	9 (4.2%)	24 (2.1%)	0.684

Data are expressed as mean (SD) or number (percentage). * Significant differences between SARS-CoV-2 variants (*p* < 0.05).

## Data Availability

All data derived from the study are in the text.
